# A Combined TLR7/TLR9/GATA3 Score Can Predict Prognosis in Biliary Tract Cancer

**DOI:** 10.3390/diagnostics11091597

**Published:** 2021-09-01

**Authors:** Vittorio Branchi, Laura Esser, Corinna Boden, Azin Jafari, Jonas Henn, Philipp Lingohr, Maria A. Gonzalez-Carmona, Marc Schmitz, Tobias J. Weismüller, Christian P. Strassburg, Steffen Manekeller, Glen Kristiansen, Jörg C. Kalff, Hanno Matthaei, Marieta I. Toma

**Affiliations:** 1 Department of General, Visceral, Thoracic and Vascular Surgery, University Hospital Bonn, 53127 Bonn, Germany; vittorio.branchi@ukbonn.de (V.B.); corinna.boden@googlemail.com (C.B.); azin.jafari@ukbonn.de (A.J.); jonas.henn@ukbonn.de (J.H.); philipp.lingohr@ukbonn.de (P.L.); steffen.manekeller@ukbonn.de (S.M.); joerg.kalff@ukbonn.de (J.C.K.); 2 Institute of Pathology, University Hospital Bonn, 53127 Bonn, Germany; laura.esser@ukbonn.de (L.E.); glen.kristiansen@ukbonn.de (G.K.); 3 Department of Internal Medicine I, University Hospital Bonn, 53127 Bonn, Germany; maria.gonzalez-carmona@ukbonn.de (M.A.G.-C.); tobias.weismueller@ukbonn.de (T.J.W.); christian.strassburg@ukbonn.de (C.P.S.); 4 Institute of Immunology, Faculty of Medicine Carl Gustav Carus, TU Dresden, 01307 Dresden, Germany; marc.schmitz@tu-dresden.de; 5 National Center for Tumor Diseases (NCT), Partner Site Dresden, 01307 Dresden, Germany; 6 German Cancer Consortium (DKTK), Partner Site Dresden, German Cancer Research Center (DKFZ), 69120 Heidelberg, Germany

**Keywords:** cholangiocarcinoma, biliary tract cancer, Toll-like receptor, GATA3

## Abstract

Biliary tract cancer (BTC) refers to a heterogenous group of epithelial malignancies arising along the biliary tree. The highly aggressive nature combined with its silent presentation contribute to the dismal prognosis of this tumor. Tumor-infiltrating immune cells (TIICs) are frequently present in BTC and there is growing evidence regarding their role as therapeutic targets. In this study, we analyzed the immune cell infiltration in BTC and developed a promising immune signature score to predict prognosis in BTC. Immunohistochemistry (IHC) was carried out on tissue microarray sections from 45 patients with resectable cholangiocarcinoma for the detection of 6-sulfoLacNAc+ monocytes (slanMo), BDCA-2+ plasmacytoid dendritic cells (pDC), CD8+ or CD4+T-lymphocytes, CD103+ cells, GATA3+ cells, Toll-like receptor (TLR) 3, 7 and 9-expressing cells as well as programmed cell death protein 1 and programmed cell death ligand 1 positive cells. Data from the IHC staining were analyzed and correlated with clinicopathological and survival data. High expression of TLR7, TLR9, and GATA3 was associated with improved overall survival (OS, Log-rank *p* < 0.05). In addition, TLR9 was associated with better disease-free survival (Log-rank *p* < 0.05). In the multivariate Cox proportional-hazards model for OS, the TLR/TLR9/GATA3 score was found to be an independent prognostic factor for OS (“Score 2” vs. “Score 0”: HR 11.17 95% CI 2.27–54.95, *p* < 0.01).

## 1. Introduction

Biliary tract cancer (BTC) comprises a diverse cluster of malignancies that can arise anywhere along the biliary tree, including the gallbladder (gallbladder carcinomas, GBC). This highly heterogeneous group of tumors occurs after a malignant transformation of various cells lining the biliary tract [1]. BTC is traditionally classified by anatomical localization as intrahepatic cholangiocarcinoma (iBTC), extrahepatic cholangiocarcinoma (eBTC) and gallbladder cancer. Extrahepatic cholangiocarcinoma can be further subdivided into perihilar (pBTC) and distal cholangiocarcinoma (dBTC) [2].

In Western countries, BTC occurs in 1–2 cases per 100,000 people, whereas in some Eastern countries the incidence is as high as 85 per 100,000 people [3]. This discrepancy can be partially explained by the geographical distribution of certain risk factors, such as parasitic infections [4,5]. However, many patients with BTC do not have known risk factors [2,6].

These cancers have a dismal prognosis due to their aggressiveness and late diagnosis which severely compromises the effectiveness of available therapeutic options [7]. Surgical resection continues to be the mainstay of curative therapy for localized disease. Locoregional lymphadenectomy, although recommended, seems to have a limited impact on overall survival (OS) [8]. Unfortunately, only about a third of patients with BTC presents with resectable disease at the time of diagnosis. In a small selected cohort of patients with iBTC, liver transplantation represents an alternative strategy to resection [9]. The current therapeutic regimens for unresectable or metastatic disease are limited and systemic chemotherapy is the main form of treatment available. The combination of gemcitabine and cisplatin remains standard first-line therapy [10]. Ten years after the ABC-02 trial, the U.S. Food and Drug Administration (FDA) approved the first targeted therapy for previously treated patients with unresectable cholangiocarcinoma with a fibroblast growth factor receptor 2 (FGFR2) fusion or rearrangement, based on the results of the FIGHT-202 trial [11]. BTCs are characterized by an intense desmoplastic reaction and substantial immune cell infiltration, including activated T-cells, dendritic cells (DCs), macrophages, and neutrophils, along with tumor-associated fibroblasts [12]. Given the limited efficacy of standard chemotherapy and the diversity of the tumor microenvironment, several promising immune and microenvironmental regulators have been proposed as personalized targeted therapy options during the last few years [13]. The efficacy of the immune checkpoint blockade has been demonstrated in a small group of BTC patients with microsatellite instability (MSI) or MMRd (mismatch repair deficiency) [14]. However, only in 1–2% of BTC a MSI or MMRd is found [15,16]. Programmed cell death ligand 1 (PD-L1) expression in BTC is variable, and several studies have addressed the utility of PD-L1 in the context of immunotherapy. Some studies suggested its role as a predictive biomarker for PD-L1 and programmed cell death protein 1 (PD-1) blockade immunotherapy [17]. Despite some promising results, the predictive role of PD-L1 in BTC remains unclear and there is actually no strong evidence that PD-L1 is a reliable biomarker for response prediction [18].

The prognosis of BTC after surgical resection is still burdened by a relapse rate ranging from 50% to 70% [19,20]. Due to the lack of efficient predictive and prognostic biomarkers in BTC, the identification of novel biomarkers to stratify BTC is pivotal. In this study, we aim to provide a deeper understanding of the possible clinical impact of immune cell infiltration in BTC.

## 2. Methods

### 2.1. Patients and Tissues

Patients who underwent surgical resection of a biliary tract cancer at the Department of Surgery, Bonn University Hospital, between 2013 and 2017 were included in this study. Patients’ demographic data including gender, age, as well as tumor and treatment-related data were collected. Survival data were retrieved from the patients’ records. Overall survival (OS) was calculated from the date of surgery to the last follow-up or death. Disease-free survival (DFS) was calculated from the date of surgery to the date of tumor relapse or last follow-up. Pathological characteristics of the tumors were obtained from the clinical records. All tumors were thoroughly restaged according to the most recent TNM classification, 8th Edition [21]. The usage of archived diagnostic left-over tissues for tissue microarray (TMA) manufacturing, the analysis for research purposes, and patient data analysis study were approved by the ethics committee, Bonn University Hospital (Nr. 417/17, 3 August 2018; Nr. 233/20, 11 September 2020). The study was carried out in compliance with the Helsinki declaration.

### 2.2. Tissue Microarray Construction and Immunohistochemistry

A tissue microarray (TMA) was constructed manually. Standard hematoxylin and eosin stained 3 µm slides were obtained to identify regions with 100% tumor tissue. For each sample, four to six representative tumor cores of 1 mm diameter were transferred from original FFPE blocks to the TMA blocks. Immunohistochemistry (IHC) was carried out on TMA sections according to standardized protocols. Antibodies to 6-sulfo LacNAc (slan), BDCA-2, CD8, CD4, CD103, GATA3, Toll-like receptor 3 (TLR3), TLR7, TLR9, PD-1 and PD-L1 were used. The details of antibodies and dilutions used are summarized in Table 1. Briefly, 3 µm sections from TMA blocks were mounted on Tomo^®^ Adhesion Microscope Slides, (Matsunami Glass Ind. LTD, Osaka, Japan). The TMA sections were deparaffinized with xylene (2 × 15 min, VWR International, Fontenay-sous-Bois, France) and rehydrated using decreasing concentrations of graded ethanol (Berkel AHK, Ludwigshafen, Germany) to water (B. Braun, Melsungen, Germany). For slan monocytes (slanMo) and pDC stainings, antigen retrieval was achieved by boiling the slides in citrate buffer (Zytomed Systems GmbH, Berlin, Germany) at pH 6.0 for 20 min. The tissue samples were then stained overnight at 4 °C with either the polyclonal goat anti-BDCA-2 antibody (1:200, R&D Systems, Minneapolis, MN, USA) to evaluate pDCs or the monoclonal mouse anti-slan antibody DD2 (1:10, Institute of Immunology, Faculty of Medicine Carl Gustav Carus, TU Dresden, Dresden, Germany) to analyze slanMo. Then, tissues used for pDC staining were incubated with a mouse anti-goat antibody solution (Thermo Fisher Scientific, Rockford, IL, USA) for 60 min. Afterward, all tissues were incubated with dextran-labeled antibodies against mouse immunoglobulins (Dako, Glostrup, Denmark) for 30 min. pDCs and slanMo were visualized by the alkaline phosphatase-based EnVisionTM detection system according to the manufacturer´s instructions (Dako).

For CD4 (clone SP35, 790–4425, ready-to-use-antibody, Roche; Basel, Switzerland) and CD8 antibodies (clone C8/144B, M7103, dilution 1:50, Agilent Technologies, Santa Clara, CA, USA), antigen retrieval was achieved by boiling the slides at 99 °C in citrate buffer at pH 6.0 for 20 min. IHC was performed using the semi-automated platform Autostainer 480 S (Medac, Wedel, Germany). All supplementary reagents were provided by Medac.

For CD103 (clone EPR4, ab129202, dilution 1:50, Abcam^®^, Cambridge, UK), GATA3 (clone L50-823, CM 405B, dilution 1:50, Novus Biologicals, Centennial, CO, USA), TLR3 (clone 40C1285.6, dilution 1:50, Novus Biologicals, NBP2-24875), TLR7 (NBP2-24906, dilution 1:100, Novus Biologicals), TLR9 (NBP2-24729, dilution 1:800, Novus Biologicals), PD-L1 (clone E1L3N, 13684, dilution, 1:50, Cell Signaling Technology^®^, Danvers, MA, USA) and PD-1 (clone NAT105, ab52587, dilution 1:200, Abcam^®^, Cambridge, UK) the antigen retrieval was performed by boiling the slides at 99 °C at pH 8.0 for 20 min. IHC was conducted using semi-automated platforms (Ventana BenchMark Ultra, Roche Diagnostics, Switzerland). All supplementary reagents were provided by Ventana Medical Systems, Inc., Tucson, AZ, USA. Antibodies details are summarized in supplementary Table S1. All TMA sections were counterstained with Mayer´s hematoxylin (Merck, Darmstadt, Germany).

### 2.3. Evaluation of Immunoreactivity

Considering the expected high amount of CD4+ and CD8+ cells, a quantitative analysis was performed using Definiens Developer XD software (v.2.0.2, Munich, Germany) with an algorithm for IHC staining. The slides were digitalized with a Zeiss Mirax scanner (Carl Zeiss, Jena, Germany) and saved in MIRAX-format. All whole slide digital images were assessed for scanning artifacts. Mean values were calculated for each case. The median was used as cut-off for dichotomization in “high CD4+/CD8+” and “low CD4+/CD8+” groups. Regarding PD-1 and PD-L1 staining, tumors with clusters of positive cells (>1%) were classified as “high PD-1/PD-L1”, whereas tumors without clusters of positive cells were classified as “low PD-1/PD-L1”. Tumor samples with infiltrating slan+ cells were classified as “slanMo+” and tumor samples without positive cells were considered as “slanMo-”. CD103 and BDCA-2 staining was evaluated by means of the percentage of positive cells in a TMA core and samples where then classified as “high/low CD103/ BDCA-2”. Mean values were calculated for each tumor. For GATA3 quantification, positively stained cells were counted for each core and the mean value was calculated for each case. Tumor samples were then dichotomized in “high GATA3” or “low GATA3” staining according to the median values. For TLR3 and TLR9 staining, tumor cells positivity was evaluated. Samples with moderate or high positive tumor cells were classified as “high TLR3” or “high TLR9”, respectively. For TLR7, samples with TLR7+ infiltrating immune cells were classified as “high TLR7”. Samples with only faintly positive cells or a negative reaction were classified as “low TLR3”, “low TLR7” or “low TLR9”.

### 2.4. Statistical Analysis

Statistical Analysis was performed with SPSS Statistics Version 22 (IBM, Armonk, New York, USA), and in the R environment (RStudio Version 1.3, package survminer version 0.4.8) [22,23]. The primary statistical objective of this study was to evaluate the prognostic value of immune cell subpopulations in BTC by means of protein expression. The Kaplan–Meier method was applied to estimate the event-time distributions for OS and DFS. Kaplan–Meier curves were compared using the log-rank method. Univariate analysis for OS and DFS was performed using the Cox proportional-hazards regression method. Parameters comparison between groups was made with Fisher’s exact test or Anova test. A *p* < 0.05 was considered statistically significant.

### 2.5. Immune Marker Score and Multivariate Analysis for OS and DFS

The IHC biomarkers with the best prognostic value for OS (TLR7, TLR9, and GATA3) were combined in an immune marker score. The score was calculated as follows: cases with negative IHC staining for TLR7, with a low TLR9 and low GATA3 positivity were classified as negative (score 0). Samples with positive IHC staining forTLR7, or TLR9 high positivity and a GATA3 positivity were classified as low positive with a score of 1 (two positive markers). Samples with a positivity for TLR7, high positivity for TLR9 and GATA3 were classified as high positive and a score of 2 was given to these samples (three positive markers). Three patients were excluded from the analysis due to missing markers due to tissue fragmentation. A multivariate Cox proportional-hazards model for OS was then used to evaluate the prognostic value of the immune marker prognostic score adjusted for significant prognostic clinical factors. All clinical factors that were prognostic when considered alone (*p* < 0.05) were added to the multivariate model for OS. A multivariate Cox proportional-hazards model for DFS was then applied. The IHC biomarker with prognostic value for DFS (TLR9) was used as the only marker score for the multivariate Cox proportional-hazards model for DFS. All clinical factors that were prognostic when considered alone (*p* < 0.05) were added to the multivariate model for DFS.

## 3. Results

### 3.1. Clinicopathological Characteristics of the Cohort

Samples from 45 patients (22 females, 23 males; median age 67, range 38–81) were analyzed. The most represented entity was iBTC (38%, *n* = 17). pBTC was diagnosed in 11 patients (24%). dBTC and GBC were diagnosed in 27% (*n* = 12) and 11% (*n* = 5) of the patients, respectively. Adjuvant chemotherapy was administered postoperatively in 58% of the patients (*n* = 26) (Table 2). Most of the tumors were T1 or T2 (*n* = 28, 62%). A positive nodal status (N1) was found in 53% (*n* = 24). Four patients had peritoneal carcinosis, which was detected after pathological examination (*n* = 4, 9%). Most of the tumors were moderately differentiated (G2, 64%, *n* = 29), and the majority did not present lymphatic invasion (L0, 76%, *n* = 34). Vascular invasion was present in 16% of the lesions (*n* = 7). In 53%, a perineurial invasion was detected (*n* = 24). Eleven patients showed microscopically positive margins after resection. The clinicopathological characteristics of the cohort are summarized in Table 1.

### 3.2. Characterization of the Immune Cell Infiltrate in BTC

**Figure 1:**
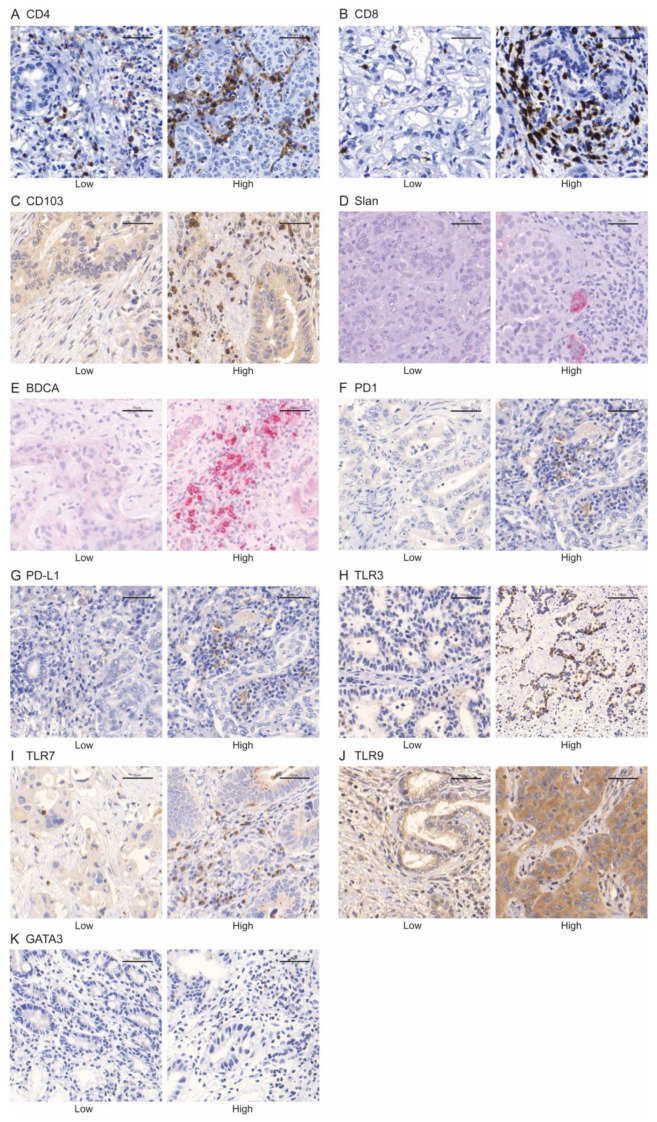
Representative images of IHC staining for CD4 (**A**), CD8 (**B**), CD103 (**C**), slan (**D**), BDCA-2 (**E**), PD1 (**F**), PD-L1 (**G**), TLR3 (**H**), TLR7 (**I**), TLR9 (**J**), and GATA3 (**K**). The scale bar represents 50 μm.

Median CD4+ cell density was 652 cells/mm^2^ (range 17–4314 cells/mm^2^) whereas median CD8+ cell density was 125 cells/mm^2^ (range 2–2216 cells/mm^2^). Median percentage of CD103+ cells was 5% (range 0–26%). In the majority of the samples (*n* = 26, 58%) an infiltration of Slan-Mo was found. Median percentage of positive BCDA+ cells was 1% (range 0–9%). In 7% of the probes (*n* = 3), clusters of PD-1+ lymphocytes were detected. In about half of the samples (52%, *n* = 23), clusters of PD-L1+ cells were observed. Most of the samples were classified as “high TLR3” (83%, *n* = 35), whereas 53% (*n* = 24) and 91% (*n* = 39) of the samples were classified as “high TLR7” and “high TLR9”, respectively. Representative examples of immunohistochemistry staining are displayed in Figure 1.

### 3.3. Immune Marker Expression and Tumor Localization

**Figure 2:**
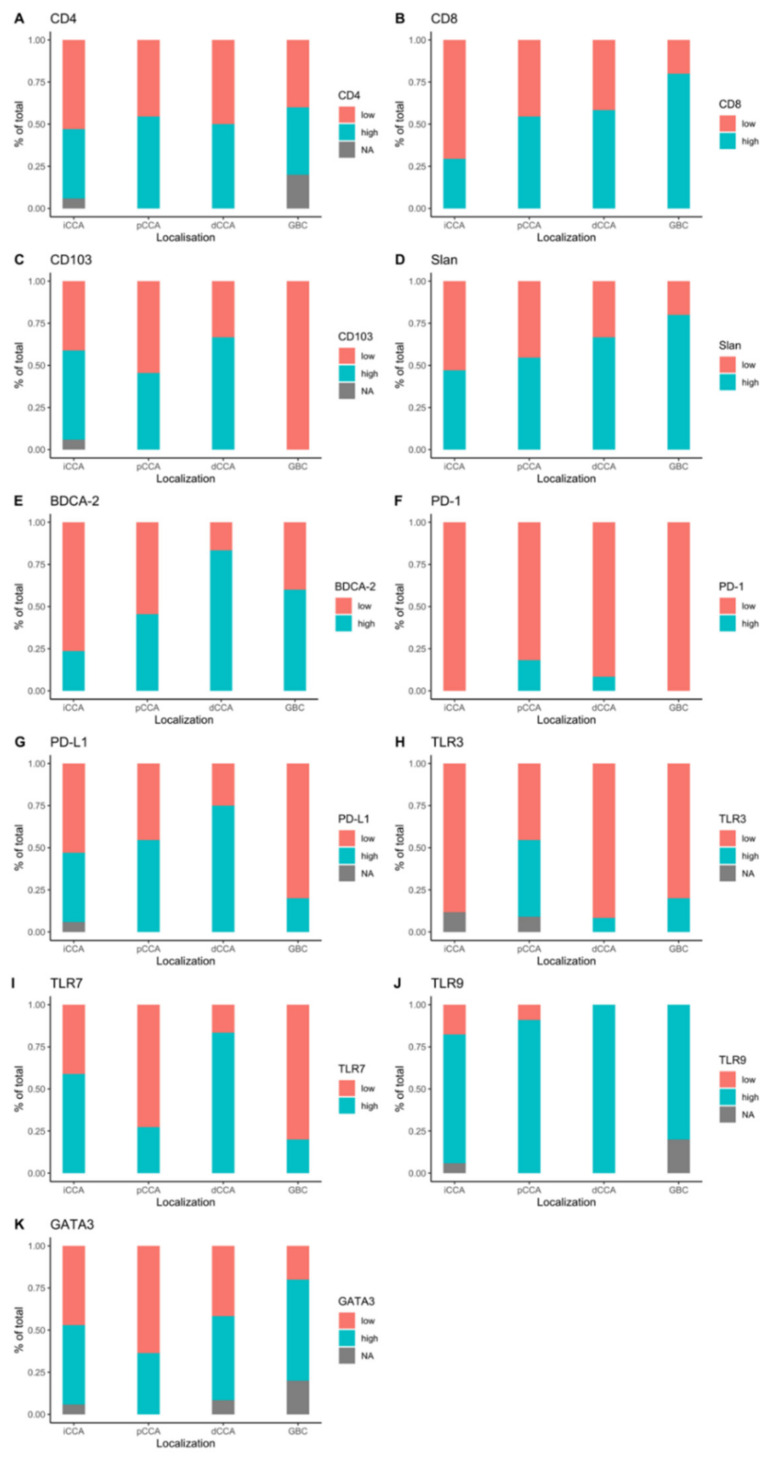
Expression of IHC markers according to tumor localization for CD4 (**A**), CD8 (**B**), CD103 (**C**), slan (**D**), BDCA-2 (**E**), PD1 (**F**), PD-L1 (**G**), TLR3 (**H**), TLR7 (**I**), TLR9 (**J**), and GATA3 (**K**). (iCCA, intrahepatic cholangiocarcinoma; pCCA, perihilar cholangiocarcinoma; dCCA, distal cholangiocarcinoma; GBC, galbladder cancer).

The expression of BDCA-2, CD103, and TLR7 was heterogeneous in different tumor localizations. In particular, dBTC had significantly more BDCA-2+ cells than iBTC and pBTC (*n* = 10, 83% vs. *n* = 4, 23% vs. *n* = 5, 45%; *p* < 0.05). Interestingly, in all samples from patients with GBC, a high expression of CD103 was observed, and the difference in expression pattern was significant compared to dBTC (*n* = 5, 100% vs. *n* = 4, 33%). The percentage of samples with high TLR7 expression was significantly higher in dBTC compared to pBTC and GBC (*n* = 10, 83% vs. *n* = 3, 27% and *n* = 1, 20%). For slan, CD4, CD8, TLR9, PD-1, PD-L1, and GATA3 no difference regarding expression and tumor localization was observed (Figure 2).

### 3.4. Immune Marker Expression and Survival

**Figure 3:**
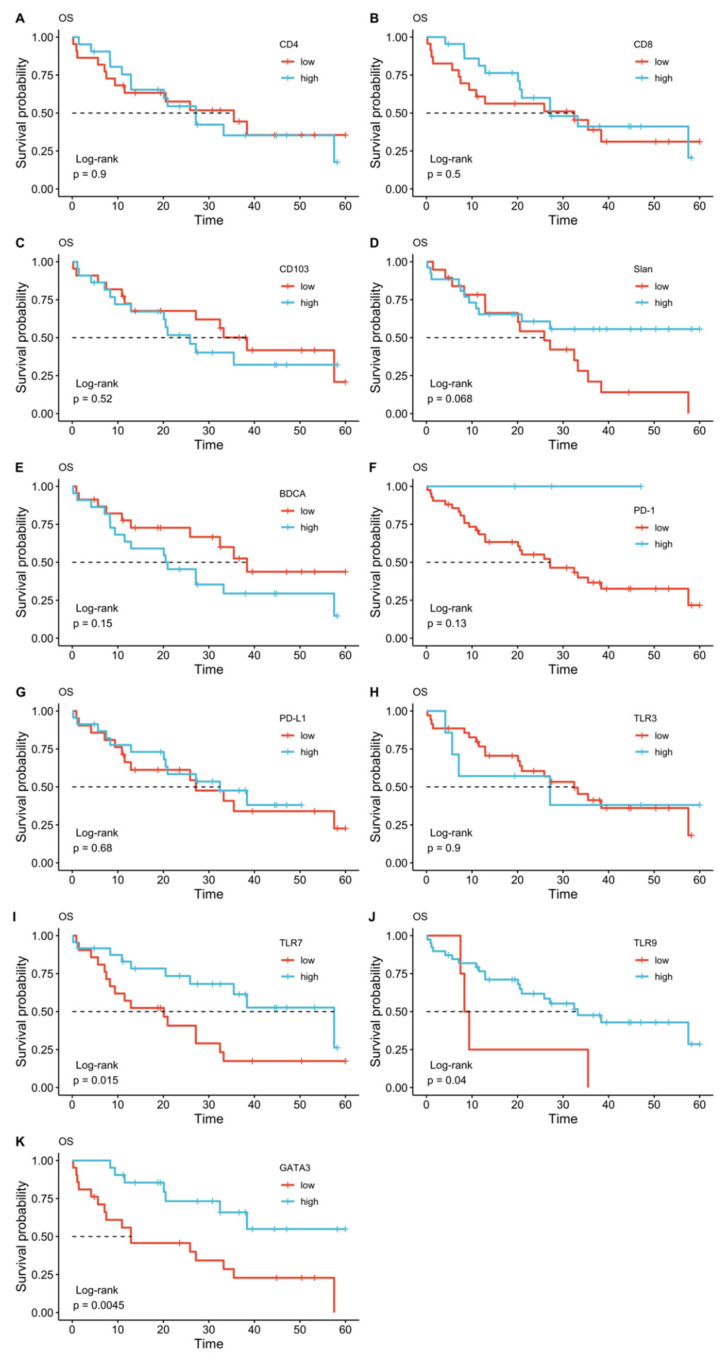
Kaplan–Meier plots for overall survival (OS) according to CD4 (**A**), CD8 (**B**), CD103 (**C**), slan (**D**), BDCA-2 (**E**), PD1 (**F**), PD-L1 (**G**), TLR3 (**H**), TLR7 (**I**), TLR9 (**J**), and GATA3 (**K**) expression.

**Figure 4:**
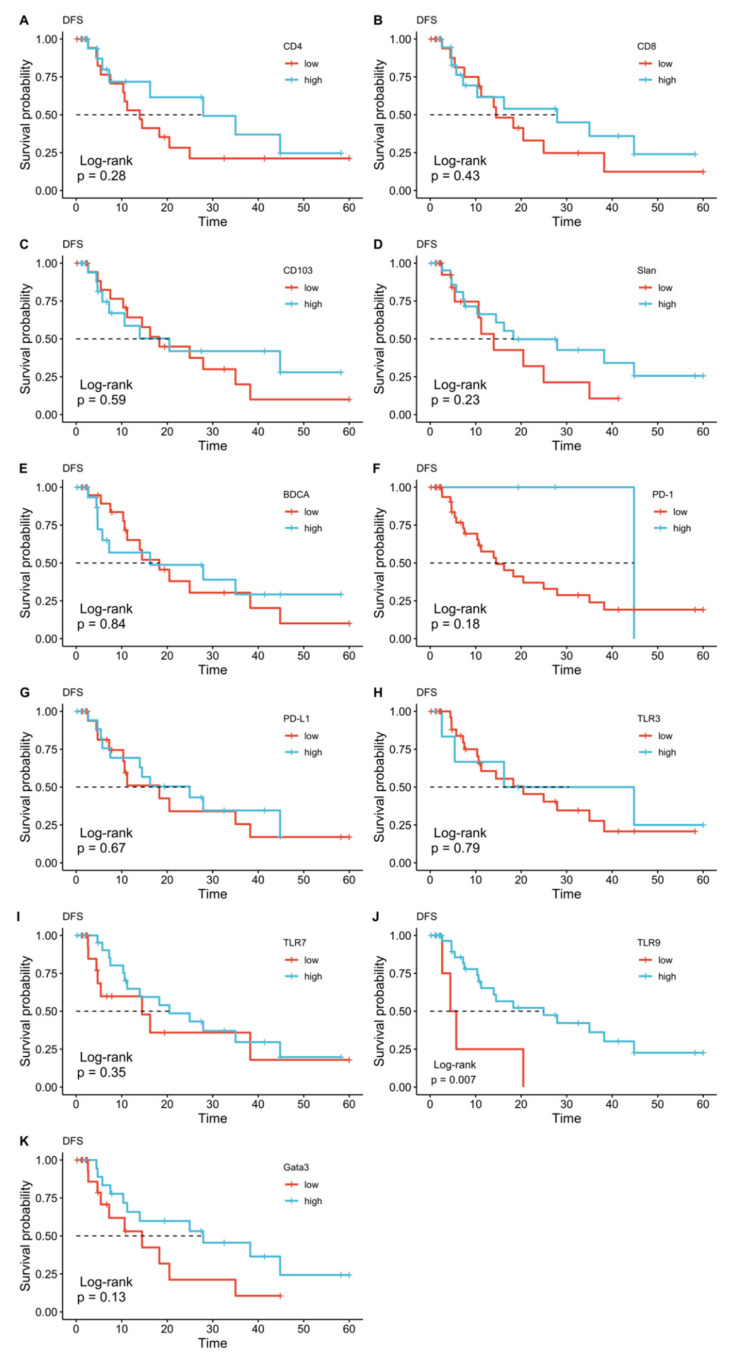
Kaplan–Meier plots for disease-free survival (DFS) according to CD4 (**A**), CD8 (**B**), CD103 (**C**), slan (**D**), BDCA-2 (**E**), PD1 (**F**), PD-L1 (**G**), TLR3 (**H**), TLR7 (**I**), TLR9 (**J**), and GATA3 (**K**) expression.

The survival analysis demonstrated that a high expression of TLR7, TLR9, and GATA3 was associated with longer overall survival. In particular, median OS was 57.5 months (95% CI 33.3–81.6 months) in the group with high TLR7 expression versus 20.1 months (95% CI 7.5–32.6 months) in the low TLR7 expression group (log-rank *p* = 0.015). In the group with high TLR9 expression, the median OS was 33.2 months (95% CI 17.6–49.0 months), whereas the group with low TLR9 expression displayed an OS of 8.3 months (95% CI 6.5–10.2 months, log-rank *p* = 0.040). Regarding GATA3 expression, median survival was not reached by the high expression group, whereas in the low expression group, the median OS was 12.9 months (95% CI 0–33.5 months, log-rank *p* = 0.004). Low expression/negativity of TLR9 was also associated with shorter DFS. In the group with high expression of TLR9, median DFS was 24.9 months (95% CI 6.1–43.7 months), and 4.4 months (95% CI 1.4–7.5 months), in the low-expression group (log-rank *p* = 0.007). (Figure 3 and Figure 4).

### 3.5. Prognostic Value of Immune Cell Infiltration Markers

The univariate Cox regression analysis for OS indicated that a high expression of TLR7,9 and GATA3 was positively correlated with a better prognosis in BTC patients. In particular, a high expression of TLR7 was significantly associated with longer OS compared to low TLR7 expression (“high TLR7” vs. “low TLR7”: HR 0.39, 95% CI 0.17–0.86, *p* = 0.020). A similar yet not statistically significant trend was found between low TLR9 expression vs. high TLR9 expression (“high TLR9” vs. “low TLR9”: HR 0.33, 95% CI 0.11–1.00, *p* = 0.050). A high GATA3 expression was also significantly correlated with a better prognosis (“high GATA3” vs. “low GATA3”: HR 0.30, 95% CI 0.12–0.72, *p* = 0.007). In addition, the univariate analysis showed that Stage III tumors were significantly associated with worse OS when compared to stage I tumors (HR 4.82, 95% CI 1.08–21.64, *p* = 0.040) (Table 2).

The results of the univariate analysis for DFS demonstrated a correlation between higher TLR9 expression and longer DFS (“high TLR9” vs. “low TLR9”: HR 0.23, 95% CI 0.07–0.74, *p* = 0.013). Among the clinicopathological parameters, a positive R status and a positive vascular invasion were significantly associated with worse DFS (R1 vs. R0: HR 5.45, 95% CI 1.89–15.71, *p* = 0.002. V1 vs. V0 HR 3.87, 95% CI 1.40–10.70, *p* = 0.009) (Table 3).

### 3.6. TLR7/TLR9/GATA3 Score and Multivariate Analysis for OS and DFS

A score was calculated using the three markers with the best prognostic significance for overall survival (TLR7, TLR9, GATA3), as described in the Methods section. Due to tissue fragmentation in at least one IHC staining, a score could not be calculated for three samples. The patients were then excluded from the analysis. Clinicopathological characteristics of the patients based on the score subgroups are summarized in Table 4.

**Figure 5:**
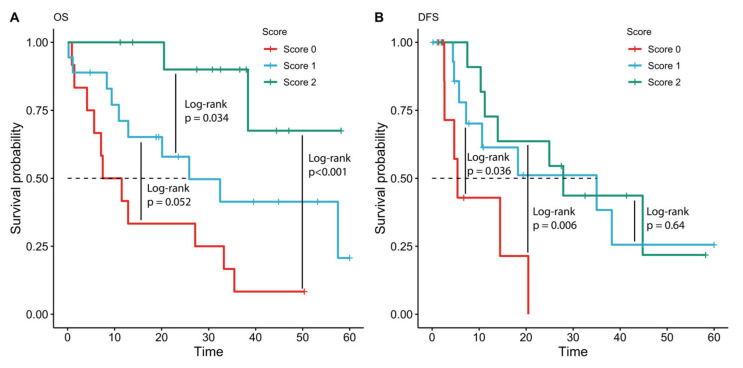
Kaplan–Meier plots for overall survival (OS, Panel (**A**)) and disease-free survival (DFS, Panel (**B**)) according to TLR7/TLR9/GATA3 score. Time in months.

The median OS in the “score 2” group was not reached, whereas in the “score 1” group the median OS was 25.9 months (95% CI 6.8–44.9 months), and 7 months (95% CI 0.0–14.9 months) in the group with a score of 0 (log-rank *p* < 0.01). The median DFS of the “score 2” group was higher than in the “score 0” group (27.9 months, 95% CI 6.8–49.0 months vs. 5.4 months, 95% CI 3.6–7.2 months; Log-rank *p* = 0.006). The median DFS in the “score 1” group was 35.0 months (CI 95% 2.5–27.5 months), which was found to be significantly longer when compared to the DFS in the “score 0” group (log-rank *p* = 0.006), and similar to the DFS in the “score 2” group (log-rank *p* = 0.644). (Figure 5).

In the multivariate Cox proportional-hazards model for OS, the TLR7/TLR9/GATA3 score was found to be an independent prognostic factor for OS (“score 0” vs. “score 2”: HR 11.17, 95% CI 2.27–54.95, *p* = 0.003; “score 1” vs. “score 2”: HR 4.45, 95% CI 0.95–20.80, *p* = 0.058). In the univariate analysis for DFS, TLR9 was the only marker significantly associated with DFS and therefore the only one added to the multivariate Cox proportional-hazards model for DFS. In the multivariate analysis, TLR9 was found to be an independent prognostic factor for DFS (“high TLR9” vs. “low TLR9”: HR 0.19, 95% CI 0.06–0.67, *p* = 0.010). R status and vascular invasion were also found to be independent prognostic factors for disease-free survival (Table 5 and Table 6).

## 4. Discussion

We performed a comprehensive analysis of tumor-infiltrating immune cells in a cohort of intrahepatic and extrahepatic BTC, including innate immunity members such as TLR7, TLR9, and adaptive immunity factors such as CD4 and CD8 as well as PD-1 and PD-L1 expression at protein level. To date, little is known about immune cell infiltration in BTC, and results are contradictory. For example, Kitano et al. found a correlation between poor overall survival and high neutrophil infiltration, low CD8 levels and regulatory T-cells [24]. Other studies failed to demonstrate an association between CD8+ lymphocytes and prognosis [25]. Goeppert et al. found a positive correlation between intraepithelial tumor-infiltrating CD4+, CD8+ and Foxp3+ T-lymphocytes and longer overall survival. Moreover, the number of tumor infiltrating CD4+ and Foxp3+ lymphocytes were independent prognostic factors for survival in BTC [26]. In this study we could not find any correlation between PD1 or PD-L1 expression or CD4+ and CD8+ tumor infiltration and prognosis. This could be related to the small cohort and tumor heterogeneity. Interestingly, we noticed significantly more pDCs in dBTC compared to iBTC or pBTC. In a recent study, Hu et al. demonstrated an association between peritumoral pDC and poor prognosis in iBTC [27]. We could not verify these results since only 17 patients with iBTC were included.

Our study reveals a potential biomarker score based on protein expression of immunological markers in patients with BTC. The score combines the expression of TLR7, TLR9, and GATA3 revealed by IHC staining of the resected samples.

TLRs belong to the evolutionarily conserved family of pattern recognition receptors (PRR) [28] that are key elements of the innate immune response [29]. They sense the presence of pathogen-associated molecular patterns (PAMPs) and danger-associated molecular patterns (DAMPS) [30]. Cell surface TLRs (TLR1, TLR2, TLR4, TLR5, TLR6, and TLR10) recognize microbial membrane lipids, whereas endosomal TLRs (TLR3, TLR7, TLR8, TLR9) detect pathogen and host-derived nucleotides [31]. In addition to their defending role against pathogens, TLRs have both tumorigenic and anti-tumor effects in cancer [32]. TLRs may promote carcinogenesis through proinflammatory, anti-apoptotic, proliferative, and profibrogenic signals in either the tumor microenvironment (TME) or tumor cells themselves [30]. TLR7 agonists have been extensively investigated over the past few years for their antitumoral activity. They promote tumor cell-killing by reverting the tumor-associated immunosuppression [33]. The mechanisms underlying the ability of TLR7 agonists to support an antitumoral response are highly diverse, ranging from IFNa secretion by DCs to natural killer (NK) cell activation [34]. Imiquimod, the only TLR7-agonist approved by the FDA and the European Medical Agency (EMA), has been successfully used for cancer immunotherapy, in particular in certain cutaneous tumors [35]. Despite the antitumor effects of TLR7 activation, recent studies underlined its potential tumor-promoting role. Ochi et al. demonstrated a high TLR7 expression in both epithelial and inflammatory cells in the context of pancreatic cancer. Besides, the authors found that a TLR7 activation leads to an acceleration of tumor formation, and pharmacological inhibition of TLR7 was associated with decreased tumor growth [36]. TLR7 is also expressed in adenocarcinoma and squamous-cell carcinoma of the lung and promotes cancer cell survival through NF-κB activation and upregulation of Bcl-2 [37].

Various studies have underlined the antitumoral function of TLR9 in cancer. The anti-tumor effect of TLR9 signals is derived from the enhanced secretion of type-1 IFN, including IFNa from pDCs through TLR9 activation [38]. However, TLR9 has also been shown to exhibit a tumor-promoting activity. For example, TLR9 signaling has been associated with an enhanced metastatic potential of lung cancer cells [39]. Conversely, TLR9 expression was linked to an angiogenic phenotype, cancer progression, and worse survival in carcinoma of the lung [40]. Furthermore, TLR9 overexpression in triple-negative breast cancer leads to epithelial-to-mesenchymal transition (EMT) induction and EGFR pathway deregulation, suggesting a role in the carcinogenesis of this tumor subtype [41]. Despite their promising role as therapeutic targets for immune therapy, TLRs have not been extensively investigated in the context of BTC yet. In our study, we demonstrate that both TLR7 and TLR9 expression are associated with a favorable prognosis in our cohort of cholangiocarcinoma patients. High TLR9 expression was also positively correlated with longer DFS. On the contrary, the expression of another member of the Toll-like receptor family, TLR3, was not associated with a survival benefit.

GATA3 belongs to a family of tissue-specific transcription factors regulating multiple developmental pathways [42]. In T cells, GATA3 plays an important in early T-cell development, from T cell commitment to differentiation and can be easily detected in developing and mature T cells and NK cells [43,44]. Some studies have shown that GATA3 is expressed in many epithelial and mesenchymal tumors [45]. In particular, GATA3 expression was linked to a favorable prognosis in lung adenocarcinoma, urothelial cancer, and breast cancer [46,47,48]. However, the prognosis relevance of GATA3 in breast cancer and other malignancies remains controversial due to inconclusive results [49]. In our cohort, GATA3 expression showed an association with OS. In the subgroup with higher GATA3+ cells, OS was significantly longer than in the group with low expression.

Our study has some limitations. First, our monocentric cohort was too small to build a validation cohort. A multicentric study with larger cohorts could address this limitation in the future. In addition, this study implied some intrinsic limitations due to its retrospective design and the results must be interpreted accordingly. Based on our findings, we introduce a novel IHC-based score that could help to stratify patients with BTC. Moreover, we demonstrated that a TLR9 signature could help to identify patients with higher risk of recurrence after resection for BTC.

## Figures and Tables

**Table 1:** Patient characteristics.

Age (%)	≤67	24 (53.3%)
	>67	21 (46.7%)
Sex (%)	Male	23 (51.1%)
	Female	22 (48.9%)
Localisation (%)	iBTC	17 (37.8%)
	pBTC	11 (24.4%)
	dBTC	12 (26.7%)
	GBC	5 (11.1%)
T (%)	T1	13 (28.9%)
	T2	15 (33.3%)
	T3	17 (37.8%)
	T4	0 (0.0%)
N (%)	N0	21 (46.7%)
	N+	24 (53.3%)
M (%)	M0	41 (91.1%)
	M1	4 (8.9%)
G (%)	G1	2 (4.4%)
	G2	29 (64.4%)
	G3	14 (31.1%)
L (%)	L0	34 (75.6%)
	L1	11 (24.4%)
V (%)	V0	38 (84.4%)
	V1	7 (15.6%)
Pn (%)	Pn0	21 (46.7%)
	Pn1	24 (53.3%)
UICC Stage (%)	Stage I	9 (20.0%)
	Stage II	16 (35.6%)
	Stage III	15 (33.3%)
	Stage IV	5 (11.1%)
R (%)	R0	34 (75.6%)
	R+	11 (24.4%)
Adjuvant Chemotherapy (%)	No	19 (42.2%)
	Yes	26 (57.8%)

G, histopathological grading; L, lymphatic invasion; V, venous invasion; Pn, Perineural invasion; UICC, Union for International Cancer Control; R, residual tumor.

**Table 2:** Univariate Cox regression analysis for overall survival (OS).

ENDPOINT		HR	CI 95%	*p*
Overall survival	Age > 67 vs. ≤67	0.96	0.44–2.11	0.917
	Female vs. Male	1.18	0.54–2.56	0.683
	T2 vs. T1	0.94	0.32–2.72	0.904
	T3 vs. T1	1.42	0.56–3.62	0.465
	N1 vs. N0	1.88	0.85–4.15	0.121
	M1 vs. M0	1.01	0.24–4.33	0.984
	Stage II vs. Stage I	3.32	0.72–15.23	0.123
	Stage III vs. Stage I	4.82	1.08–21.64	0.040
	Stage IV vs. Stage I	3.04	0.43–21.78	0.267
	G1-2 vs. G3	1.00	0.42–2.40	0.999
	R1 vs. R0	1.24	0.49–3.14	0.658
	V1 vs. V0	1.77	0.66–4.73	0.257
	L1 vs. L0	1.02	0.41–2.55	0.970
	Pn1 vs. Pn0	1.53	0.70–3.36	0.283
	Chemotherapy	0.72	0.33–1.57	0.410
	PD1 high vs. low	23.01	0.05–11,831.45	0.325
	PD-L1 high vs. low	0.84	0.38–1.88	0.677
	CD103 high vs. low	1.30	0.59–2.86	0.520
	Slan high vs. low	0.49	0.23–1.07	0.075
	BDCA-2 high vs. low	1.79	0.81–3.96	0.151
	TLR3 high vs. low	1.07	0.36–3.16	0.900
	TLR7 high vs. low	0.39	0.17–0.86	0.020
	TLR9 high vs. low	0.33	0.11–1.00	0.050
	GATA3 high vs. low	0.30	0.12–0.72	0.007
	CD4 high vs. low	1.05	0.48–2.31	0.898
	CD8 high vs. low	0.77	0.35–1.66	0.501

HR, Hazard ratio; CI, confidence Interval; G, histopathological grading; L, lymphatic invasion; V, venous invasion; Pn, Perineural invasion; UICC, Union for International Cancer Control; R, residual tumor.

**Table 3:** Univariate Cox regression analysis for disease-free survival (DFS).

ENDPOINT.		HR	CI 95%	*p*
Disease free survival	Age >67 vs. ≤67	0.73	0.30–1.74	0.478
	Female vs. Male	0.71	0.30–1.66	0.426
	T2 vs. T1	0.46	0.15–1.36	0.160
	T3 vs. T1	0.71	0.26–1.91	0.498
	N+ vs. N0	1.24	0.53–2.92	0.616
	M1 vs. M0	1.64	0.48–5.63	0.429
	Stage II vs. Stage I	0.80	0.24–2.63	0.709
	Stage III vs. Stage I	1.25	0.39–3.94	0.707
	Stage IV vs. Stage I	1.51	0.36–6.42	0.576
	G1-2 vs. G3	1.17	0.49–2.81	0.724
	R1 vs. R0	5.45	1.89–15.71	0.002
	V1 vs. V0	3.87	1.40–10.70	0.009
	L1 vs. L0	1.51	0.60–3.81	0.379
	Pn1 vs. Pn0	0.98	0.42–2.30	0.966
	Chemotherapy	1.22	0.49–3.00	0.667
	PD-L1 high vs. low	0.83	0.36–1.93	0.672
	PD1 high vs. low	0.27	0.04–2.05	0.208
	CD103 high vs. low	0.79	0.33–1.87	0.591
	Slan high vs. low	0.59	0.25–1.41	0.233
	BDCA-2 high vs. low	0.92	0.39–2.16	0.840
	TLR3 high vs. low	0.86	0.28–2.62	0.791
	TLR7 high vs. low	0.66	0.28–1.59	0.357
	TLR9 high vs. low	0.23	0.07–0.74	0.013
	GATA3 high vs. low	0.51	0.22–1.23	0.134
	CD4 high vs. low	0.61	0.25–1.50	0.281
	CD8 high vs. low	0.71	0.30–1.67	0.437

HR, Hazard ratio; CI, confidence Interval; G, histopathological grading; L, lymphatic invasion; V, venous invasion; Pn, Perineural invasion; UICC, Union for International Cancer Control; R, residual tumor.

**Table 4:** Patients’ characteristics in different subgroups, according to IHC score.

		Score 0	Score 1	Score 2	*p*
Number of patients		12	18	12	
Age (%)	≤67	8 (66.7%)	9 (50.0%)	6 (50.0%)	0.728
	>67	4 (33.3%)	9 (50.0%)	6 (50.0%)	
Sex (%)	Male	6 (50.0%)	8 (44.4%)	9 (75.0%)	0.324
	Female	6 (50.0%)	10 (55.6%)	3 (25.0%)	
Localisation (%)	iBTC	4 (33.3%)	8 (44.4%)	4 (33.3%)	0.24
	pBTC	5 (41.7%)	5 (27.8%)	1 (8.3%)	
	dBTC	1 (8.3%)	4 (22.2%)	6 (50.0%)	
	GBC	2 (16.7%)	1 (5.6%)	1 (8.3%)	
T (%)	T1	4 (33.3%)	5 (27.8%)	4 (33.3%)	0.032
	T2	2 (16.7%)	11 (61.1%)	2 (16.7%)	
	T3	6 (50.0%)	2 (11.1%)	6 (50.0%)	
	T4	0 (0.0%)	0 (0.0%)	0 (0.0%)	
M (%)	M0	11 (91.7%)	17 (94.4%)	11 (91.7%)	1
	M1	1 (8.3%)	1 (5.6%)	1 (8.3%)	
N (%)	N0	3 (25.0%)	11 (61.1%)	7 (58.3%)	0.163
	N+	9 (75.0%)	7 (38.9%)	5 (41.7%)	
G (%)	G1	0 (0.0%)	2 (11.1%)	0 (0.0%)	0.312
	G2	7 (58.3%)	13 (72.2%)	7 (58.3%)	
	G3	5 (41.7%)	3 (16.7%)	5 (41.7%)	
L (%)	L0	8 (66.7%)	14 (77.8%)	11 (91.7%)	0.407
	L1	4 (33.3%)	4 (22.2%)	1 (8.3%)	
V (%)	V0	8 (66.7%)	17 (94.4%)	11 (91.7%)	0.143
	V1	4 (33.3%)	1 (5.6%)	1 (8.3%)	
Pn (%)	Pn0	4 (33.3%)	9 (50.0%)	7 (58.3%)	0.46
	Pn1	8 (66.7%)	9 (50.0%)	5 (41.7%)	
UICC Stage (%)	Stage I	1 (8.3%)	4 (22.2%)	4 (33.3%)	0.102
	Stage II	2 (16.7%)	7 (38.9%)	6 (50.0%)	
	Stage III	8 (66.7%)	5 (27.8%)	1 (8.3%)	
	Stage IV	1 (8.3%)	2 (11.1%)	1 (8.3%)	
R (%)	R0	9 (75.0%)	14 (77.8%)	9 (75.0%)	1
	R+	3 (25.0%)	4 (22.2%)	3 (25.0%)	
Adjuvant Chemotherapy (%)	No	6 (50.0%)	9 (50.0%)	4 (33.3%)	0.728
	Yes	6 (50.0%)	9 (50.0%)	8 (66.7%)	

G, histopathological grading; L, lymphatic invasion; venous invasion; Pn, Perineural invasion; UICC, Union for International Cancer Control; R, residual tumor.

**Table 5:** Multivariate Cox proportional-hazards model for overall survival (OS).

Endpoint		HR	CI 95%	*p*
Overall survival	Score 0 vs. Score 2	11.17	2.27–54.95	0.003
	Score 1 vs. Score 2	4.45	0.95–20.80	0.058
	Stage II vs. Stage I	3.55	0.75–16.72	0.109
	Stage III vs. Stage I	2.80	0.61–12.92	0.186
	Stage IV vs. Stage I	1.28	0.11–14.31	0.841

**Table 6:** Multivariate Cox proportional-hazards model for disease-free survival (DFS).

Endpoint		HR	CI 95%	*p*
Disease-free survival	TLR9 high v low	0.19	0.06–0.67	0.010
	V1 vs. V0	3.78	1.15–12.43	0.028
	R1 vs. R0	3.96	1.26–12.39	0.018

## Data Availability

The data presented in this study are available upon reasonable request.

## Supplementary Materials

The following are available online at https://www.mdpi.com/article/10.3390/diagnostics11091597/s1, Table S1: Antibody details.
