# Supplementary material for: A Combined TLR7/TLR9/GATA3 Score Can Predict Prognosis in Biliary Tract Cancer
Source: Diagnostics (Basel). 2021 Sep 1;11(9):1597. doi: 10.3390/diagnostics11091597 (PMC8469358; doi:10.3390/diagnostics11091597)
Supplement: Supplementary file 1 [file diagnostics-11-01597-s001.zip › diagnostics-1268180-supplementary.pdf]

Supplementary Materials

Table S1. Antibody details.

Antybody	Clone		Diluition	Buffer	Platform
GATA3	L50-823	Biocare Medical	1:50	pH 8.0	BenchMark Ultra
TLR3	40C1285.6	Novus Biologicals	1:50	pH 8.0	BenchMark Ultra
TLR7	Polyclonal	Novus Biologicals	1:100	pH 8.0	BenchMark Ultra
TLR9	26C593.2	Novus Biologicals	1:800	pH 8.0	BenchMark Ultra
CD8	C8/144B	Agilent	1:50	pH 6.0	Autostainer
CD4	SP35	Roche	1:50	pH 6.0	Autostainer
CD103	EPR4	Abcam®	1:50	pH 8.0	BenchMark Ultra
BDCA-2	Polyclonal	R&D Systems	1:200	pH 6.0	Manual
Slan	DD2	Proprietary*	1:10	pH 6.0	Manual
PD-1	NAT105	Abcam®	1:200	pH 8.0	BenchMark Ultra
PD-L1	E1L3N	Cell Signaling Technology®	1:50	pH 8.0	BenchMark Ultra

* Institute of Immunology, Faculty of Medicine Carl Gustav Carus, Technische Universität Dresden (Dresden, Germany).
